# No effect of deleted in malignant brain tumors 1 deficiency on chemotherapy induced murine intestinal mucositis

**DOI:** 10.1038/s41598-021-94076-w

**Published:** 2021-07-19

**Authors:** Anders B. Nexoe, Andreas A. Pedersen, Sebastian von Huth, Grith L. Sorensen, Uffe Holmskov, Ping-Ping Jiang, Sönke Detlefsen, Steffen Husby, Mathias Rathe

**Affiliations:** 1 Department of Molecular Medicine, Cancer and Inflammation Research, University of Southern Denmark, Odense, Denmark; 2 Department of Gastroenterology, Odense University Hospital, Odense, Denmark; 3 Department of Infectious Diseases, Odense University Hospital, Odense, Denmark; 4 Department of Veterinary and Animal Sciences, University of Copenhagen, Copenhagen, Denmark; 5 Department of Pathology, Odense University Hospital, Odense, Denmark; 6 Hans Christian Andersen Children’s Hospital, Odense University Hospital, Sdr. Boulevard 29, 5000 Odense C, Denmark; 7 Department of Clinical Research, Faculty of Health Sciences, University of Southern Denmark, Odense, Denmark

**Keywords:** Experimental models of disease, Immunology, Medical research, Molecular medicine, Pathogenesis

## Abstract

Mucositis is a serious adverse effect of chemotherapeutic treatment. During intestinal mucositis, the mucosal barrier is compromised, increasing the risk of severe infections. Mucositis necessitates dose reduction or pauses in treatment, which affect the outcome of the treatment. Deleted in malignant brain tumors 1 (DMBT1) is a secreted scavenger protein with effects on innate immunity and epithelial regeneration. We have previously shown that jejunal DMBT1 expression is increased in piglets during chemotherapeutic treatment. We hypothesized that DMBT1 ameliorates doxorubicin-induced mucositis. Individually-caged *Dmbt1*^+/+^ (WT) and *Dmbt1*^−/−^ (KO) female mouse littermates received intraperitoneal injections of either doxorubicin or saline. They were euthanized after three (D3) or seven days (D7). Weight loss was monitored every day, and serum citrulline levels were measured at termination. Intestinal tissue was analyzed for the expression of DMBT1 and proinflammatory cytokines (IL-1β, IL-6, and TNF). Specimens from the small intestines and colon were scored for inflammation and epithelial and mucosal architecture changes. We detected no effect of DMBT1 on weight loss, serum citrulline levels, expression of proinflammatory cytokines, or histologic damage. We detected a significant increase in crypt depth in WT mice compared to that in KO mice on D3. In conclusion, DMBT1 does not affect doxorubicin-induced mucositis in mice.

## Introduction

Chemotherapy-induced mucositis is a common adverse event during cancer treatment^1,2^. Mucositis compromises nutrient absorption and the immunological barrier function of the gastrointestinal tract, which leads to apoptosis of the crypts and hypoplastic villus atrophy in the small intestine^3^. Mucositis increases the risk of infections, and mucositis may lead to dose reduction or cessation of treatment, which can reduce survival^4–6^. There is no efficient preventive or curative treatment for mucositis, and current strategies are mainly aimed at relieving the symptoms^7–9^. Identification of important targetable biomarkers related to the pathogenesis of chemotherapy-induced mucositis would be of great importance. Such biomarkers could potentially serve as a basis for improved clinical management of cancer patients in the future^10,11^.

Deleted in malignant brain tumors 1 (DMBT1), also known as salivary agglutinin (SAG), salivary scavenger and agglutinin (SALSA), glycoprotein 340 and hensin, is a secreted scavenger protein with functions in mucosal innate immunity and epithelial differentiation and regeneration^12,13^. DMBT1 binds to dextran sodium sulfate (DSS) and carrageenan but does not reduce cytotoxicity in vitro^14^, while variations in the DMBT1-encoding gene are related to Crohn’s disease^15,16^. DMBT1 has antimicrobial and antiviral properties and binds to the innate immunoproteins surfactant A and surfactant D^17–20^. Loss of DMBT1 is associated with an increased risk of colorectal, thyroid, breast, and skin cancer^21–24^. DMBT1 promotes activation of the lectin pathway when bound to the cell surface and inhibits the lectin pathway when it is in solution^25^. We have previously shown that DMBT1 expression is upregulated during chemotherapy-induced mucositis in piglets^26^.

We hypothesized that DMBT1 might confer protective effects during chemotherapy-induced mucositis. We tested this hypothesis in a murine model of doxorubicin-induced mucositis by comparing WT mice expressing DMBT1 (*Dmbt1*^+*/*+^) with DMBT1-deficient KO mice (*Dmbt1*^*−/−*^).

## Results

### Doxorubicin induces weight loss and decreases citrulline levels but does not affect intestinal length

**Figure 1:**
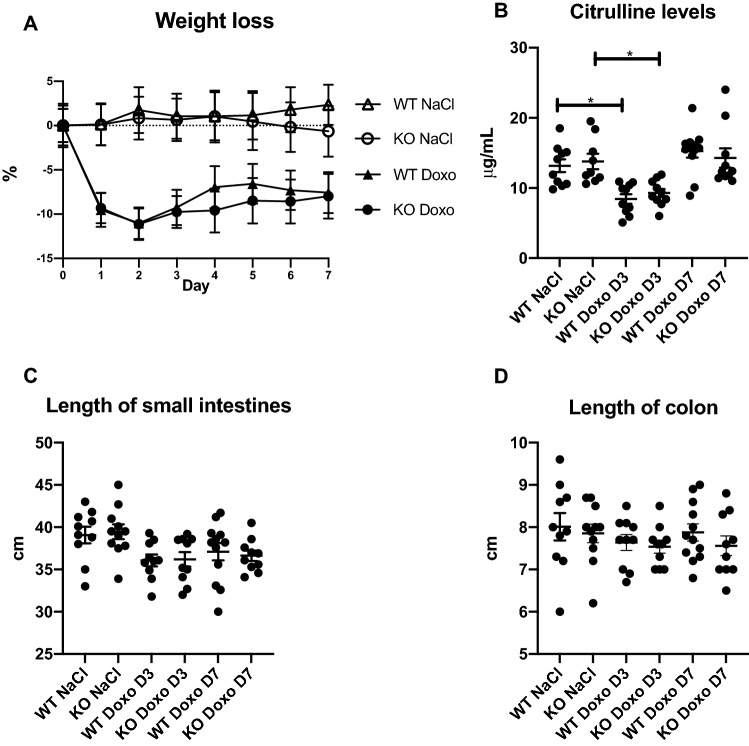
Weight loss, serum citrulline levels and intestinal length. (**A**) weight loss following doxorubicin treatment. (**B**) serum citrulline levels at termination. (**C**) length of the small intestine at termination. (**D**) length of colon at termination. All data are shown as the means ± SEM. * *p* value < 0.05.

Treatment with doxorubicin induced significant weight loss from day 0 to day 2 (WT NaCl vs WT Doxo *p* = 0.0013; KO NaCl vs KO Doxo *p* = 0.0146), which was followed by a period of stagnation/minor restitution that showed the same course in mice with different genotypes (Fig. 1A). Doxorubicin treatment induced a significant decrease in citrulline levels on D3 but not on D7 compared to saline injection (Fig. 1B. WT NaCl vs WT Doxo D3 *p* = 0.0282; KO NaCl vs KO Doxo D3 *p* = 0.0419). Since citrulline is a marker of functional enterocyte mass and absorption and gastrointestinal mucositis, these data indicate that the mice were still suffering from the effects of doxorubicin on D3 but were in the restitution phase on D7^27,28^. We detected no significant effect of doxorubicin on the length of the small intestines and colon (Fig. 1C, D).

### Induction of DMBT1 expression in the jejunum and colon following doxorubicin treatment

**Figure 2:**
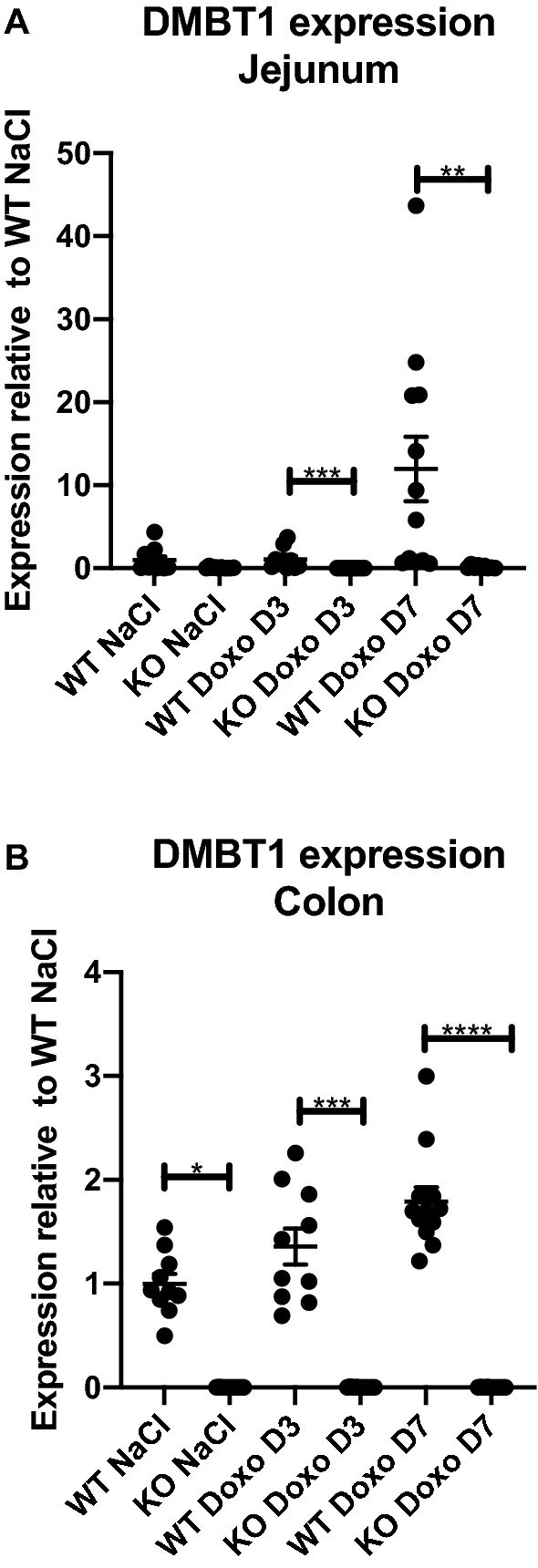
DMBT1 expression in the murine jejunum and colon following doxorubicin injection. (**A**) Jejunal DMBT1 expression. (**B**) Colonic DMBT1 expression. All data are shown as the means ± SEM. * *p* < 0.05; ** *p* < 0.01; *** *P* < 0.001; **** p < 0.0001.

There was no significant difference in jejunal DMBT1 expression between WT and KO mice before doxorubicin treatment. However, following doxorubicin treatment, we observed an increase in the induction of DMBT1 expression in WT mice compared to that in KO (Fig. 2A. WT Doxo D3 vs KO Doxo D3 *p* = 0.0003; WT Doxo D7 vs KO Doxo D7 *p* = 0.0058). In the colon, there was a significant difference in DMBT1 expression between WT and KO mice, which increased on D3 and D7 following doxorubicin treatment (Fig. 2B. WT NaCl vs KO NaCl *p* = 0.0257; WT Doxo D3 vs KO Doxo D3 *p* = 0.0005; WT Doxo D7 vs KO Doxo D7 *p* < 0.0001). The quantitative real-time polymerase chain reaction (qRT-PCR) cycle threshold (Ct) had a mean value of 27.9 for the jejunum and 20.2 for the colon in saline-treated WT mice.

**Figure 3:**
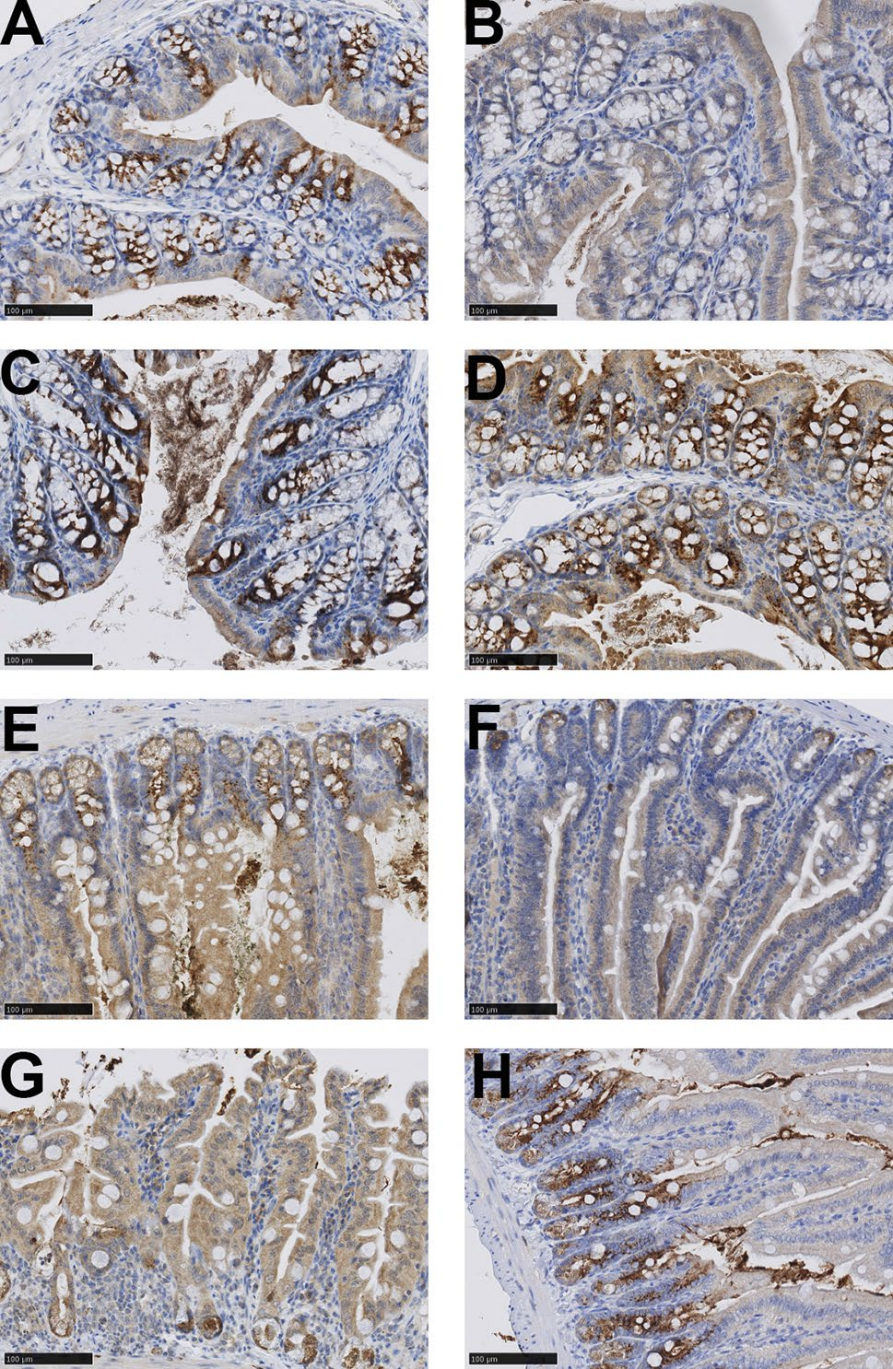
DMBT1-stained tissue. (**A**) colon from WT NaCl. (**B**) colon from KO NaCl. (**C**) colon from WT Doxo D3. (**D**) colon from WT Doxo D7. (**E**) jejunum from WT NaCl. (**F**) jejunum from KO NaCl. (**G**) jejunum from WT Doxo D3. (**H**) jejunum from WT Doxo D7. Bars indicate 100 μm.

This induction was also reflected on the protein level where the epithelial cells showed stronger staining for DMBT1 on D3 following treatment with doxorubicin in both colon and jejunum (Fig. 3). We also detected some DMBT1-staining of the Paneth cells in the jejunal crypts of KO mice (Fig. 3F).

### Induction of interleukin (IL)-1β, IL-6 and tumor necrosis factor (TNF) expression following doxorubicin treatment

**Figure 4:**
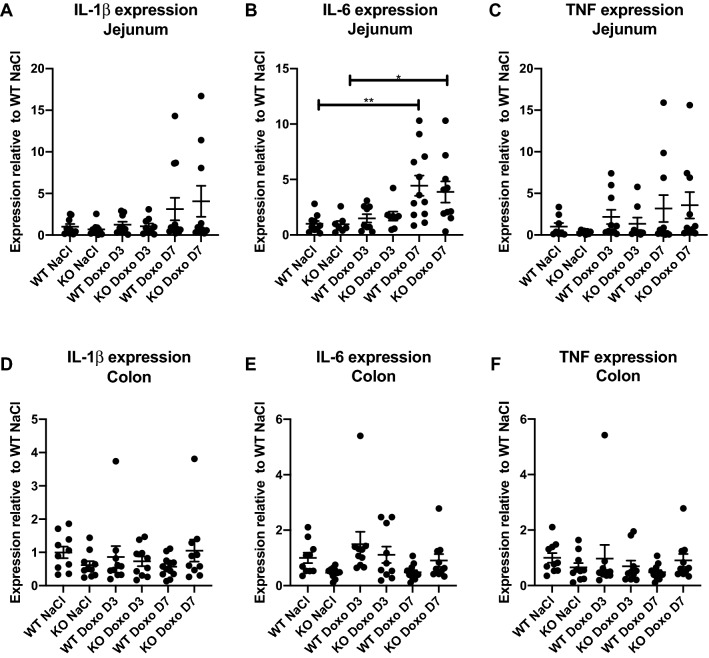
Expression of proinflammatory cytokines determined by qRT-PCR. (**A–C**) Jejunal expression of IL-1β, IL-6 and TNF. (**D–F**) Colonic expression of IL-1β, IL-6 and TNF. All data are shown as the means ± SEM. * *p* < 0.05; ** *p* < 0.01.

The proinflammatory cytokines IL-1β, IL-6, and TNF were measured by qRT-PCR analysis. We detected a significant increase in jejunal IL-6 expression in doxorubicin-treated WT and KO mice at D7 compared to that in saline-treated littermates (Fig. 4B. WT NaCl vs WT Doxo D7 *p* = 0.0042; KO NaCl vs KO Doxo D7 *p* = 0.0424), but there were no genotype-specific differences (Fig. 4).

### Doxorubicin-induced histologic damage in the jejunum but not in the colon

**Figure 5:**
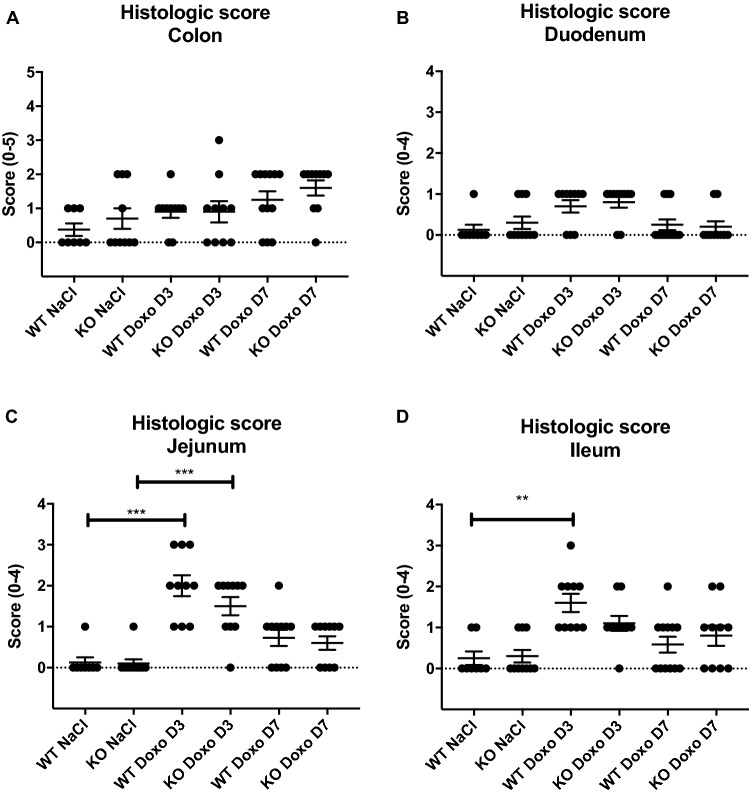
Histologic damage score. (**A**) Histologic damage score of colonic tissue. (**B**) Histologic damage score of duodenal tissue. (**C**) Histologic damage score of jejunal tissue. (**D**) Histologic score of ileal tissue. All data are presented as the means ± SEM. **p < 0.01, *** *p* < 0.001.

**Figure 6:**
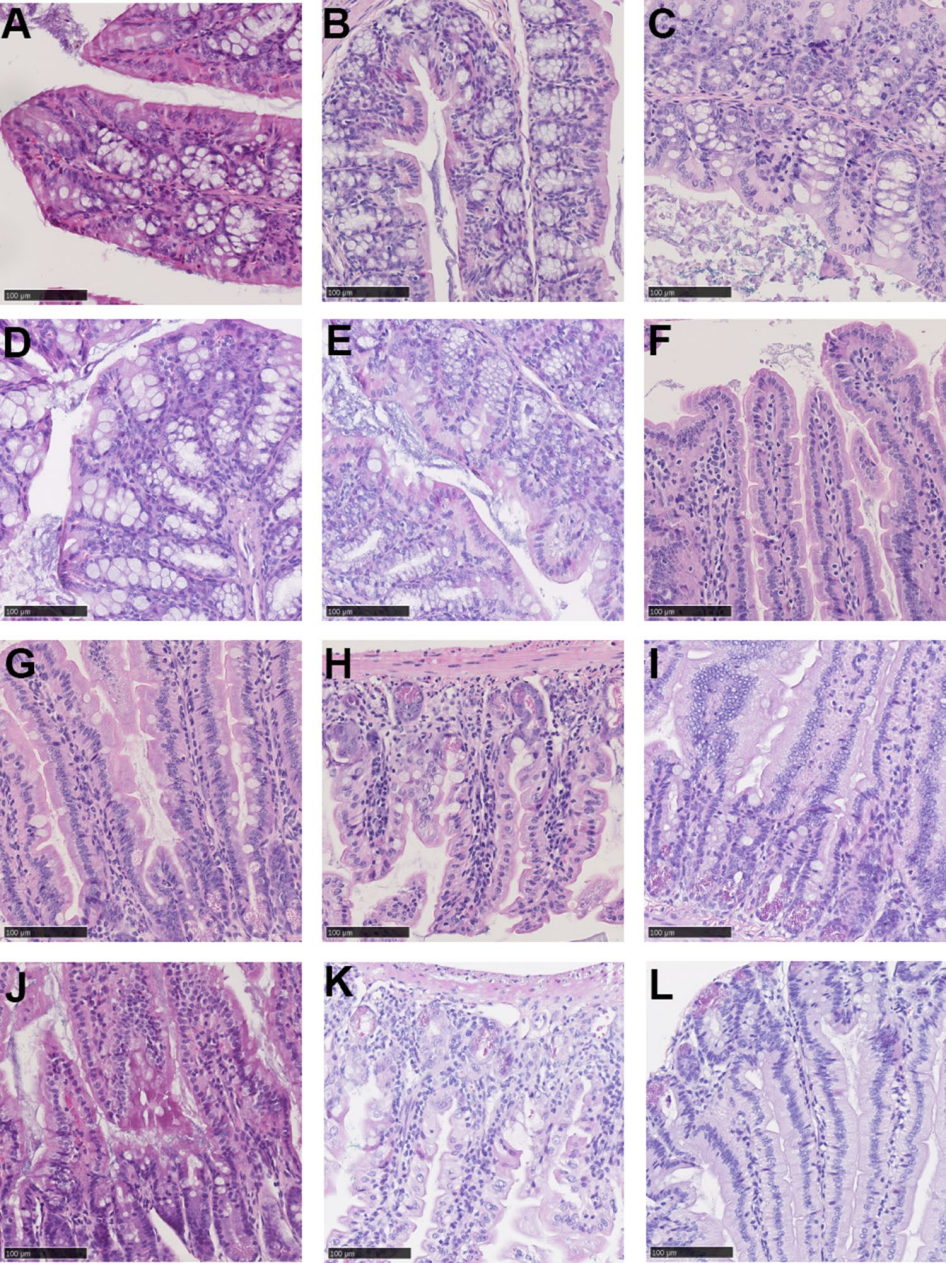
H&E-stained tissue. (**A**) colon from WT NaCl. (**B**) colon from WT Doxo D3. (**C**) colon from WT Doxo D7. (**D**) colon from KO NaCl. (**E**) colon from KO Doxo D3. (**F**) colon from KO Doxo D7. (**G**) jejunum from WT NaCl. (**H**) jejunum from WT Doxo D3. (**I**) jejunum from WT Doxo D7. (**J**) jejunum from KO NaCl. (**K**) jejunum from KO Doxo D3. (**L**) jejunum from KO Doxo D7. Bars indicate 100 μm.

Histologic damage was evaluated by examining hematoxylin and eosin (H&E)-stained colonic and small intestinal tissue. We detected no significant difference in the histologic damage score in the colonic tissue of mice subjected to saline and doxorubicin treatment (Fig. 5A). We detected no differences in histologic damage in the duodenum (Fig. 5B). In the jejunum, we detected a significant difference between the results of saline and doxorubicin treatment, as both WT and KO doxorubicin-treated mice showed increased histologic damage scores on D3 compared to mice treated with saline. There was no genotypic difference (Fig. 5C. WT NaCl vs WT Doxo D3 *p* = 0.0001; KO NaCl vs KO Doxo D3 *p* = 0.001). In the ileum there was only a significant difference between saline and doxorubicin in the WT group (Fig. 5D. WT NaCl vs WT Doxo D3 *p* = 0.0017). On D7, there were no significant differences between the mice subjected to doxorubicin and saline treatments. Figure 6 show photo microscopy of H&E stained colonic and jejunal tissue.

### DMBT1 increased the duodenal crypt depth on D3

**Figure 7:**
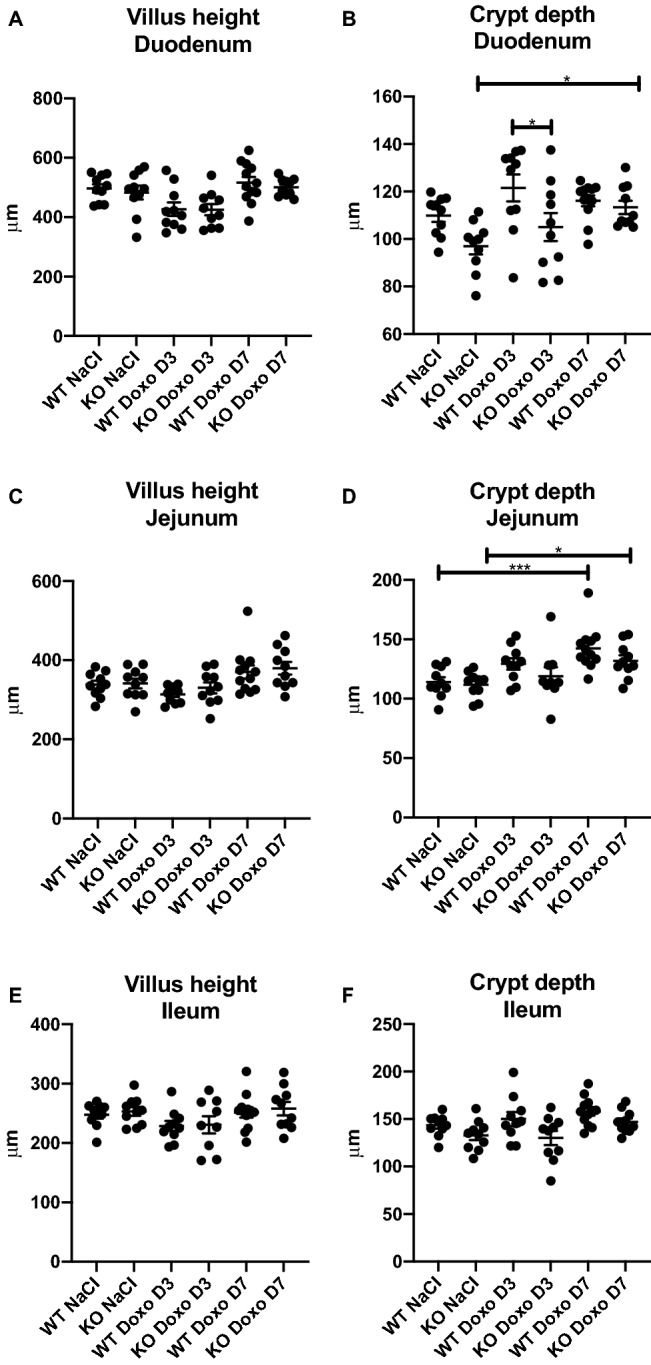
Villus height and crypt depth of small intestinal segments. (**A**) Duodenal villus height in the duodenum. (**B**) Duodenal crypt depth. (**C**) Jejunal villus height. (**D**) Jejunal crypt depth. (**E**) Ileal villus height. (**F)** Ileal crypt depth. All data are presented as the means ± SEM. * *p* < 0.05, *** *p* < 0.001.

Villus height and crypt depth were measured in H&E-stained tissue from the duodenum, jejunum, and ileum (Fig. 7). There was no difference in duodenal villus height between any of the groups (Fig. 7A), but we observed a significant increase in the duodenal crypt depth in doxorubicin-treated WT mice compared to that in doxorubicin-treated KO mice on D3 (Fig. 7B. WT Doxo D3 vs KO Doxo D3 *p* = 0.0377). In addition, we observed a significant increase in the crypt depth (Fig. 7B. KO NaCl vs KO Doxo D7 *p* = 0.0378) and an increase in the jejunal crypt depth in doxorubicin-treated KO mice compared with that in saline-treated KO mice on D7 (Fig. 7D. WT NaCl vs WT Doxo D7 *p* = 0.0009; KO NaCl vs KO Doxo D7 *p* = 0.0422). There were no differences in the villus height in the duodenum (Fig. 7A), jejunum (Fig. 7C), or ileum (Fig. 7E), or in crypt depth in the ileum (Fig. 7F).

## Discussion

In the present in vivo study, we investigated the potential ameliorating effects of DMBT1 on chemotherapy-induced intestinal mucositis. Since a previous study detected increased intestinal DMBT1 expression in chemotherapy-treated piglets^26^, and DMBT1 was shown to function in the immune system and in epithelial regeneration, we hypothesized that it would ameliorate chemotherapy-induced mucositis. However, we detected no significant effects of DMBT1 on weight loss, serum citrulline levels, intestinal length, expression of proinflammatory cytokines, or villus height.

Gastrointestinal mucositis may manifest as abdominal pains as well as diarrhea or constipation, nausea, and vomiting^2,29^. Such clinical manifestations may vary between different cytotoxic regimens.

In the current study, Doxorubicin treatment resulted a significant decrease in citrulline levels on D3, weight loss and a significant difference between the results of saline and doxorubicin treatment, as both WT and KO doxorubicin-treated mice showed increased histologic damage scores on D3 compared to mice treated with saline. All hallmarks of GI toxicity. The decreased citrulline observed at day 3 as compared to saline treated animals substantiates a relevant toxic effect on the GI mucosa^30–34^. Based on these findings we conclude that doxorubicin-treated mice are relevant for studying chemotherapy-induced gut toxicity.

We observed a significant effect of DMBT1 on duodenal crypt depth on D3, where WT mice showed an increase in the crypt depth compared to KO mice following treatment with doxorubicin. However, since this was the only genotypic difference observed in this study and DMBT1 did not affect weight loss or serum citrulline levels, the clinical significance is limited.

Although we detected a significant increase in IL-6 expression on D7 in doxorubicin-treated animals, we detected no difference in the expression of IL-1β and TNF between mice given doxorubicin or saline treatment on D3 and D7. This could suggest that inflammation was already in resolution on D3. This is in line with the histologic evaluation of H&E-stained tissue, which showed the significant effect of doxorubicin in the small intestines on D3 but not on D7, and this highlights that gastrointestinal toxicity is a highly dynamic process^26,35^.

We detected no difference in jejunal DMBT1 expression between saline WT and KO animals, suggesting the limited expression of DMBT1 in the WT animals. However, following chemotherapeutic treatment, there was a significant difference in the induction of DMBT1 expression in WT and KO mice on both D3 and D7 and a trend towards an increase in the expression of DMBT1 in WT mice. This is in line with previous observations showing that the expression of DMBT1 was upregulated in piglets during treatment with either busulfan plus cyclophosphamide or doxorubicin^26^. We also detected an increased staining of DMBT1 in intestinal epithelial cells following treatment with doxorubicin in both colon and jejunum. We also detected some minor staining of jejunal crypts in KO mice, which could suggest that the KO mice were able to express DMBT1 in a minor degree. However, the expression of DMBT1 in jejunal tissue in the KO mice was very low and there was no increase of DMBT1 expression following treatment with doxorubicin. We therefore believe that the DMBT1-staining is more likely to be due to unspecific staining or at least of minor relevance.

A recent study investigating the distribution and localization of DMBT1 in healthy human tissue found that the DMBT1 cycle threshold (Ct) values in the jejunum and colon were 17 and 22, respectively^36^. In comparison, the Ct values for saline-treated mice were 28 and 20 for the jejunum and colon, respectively. The higher expression of DMBT1 in the human intestine compared with that in the murine intestine could indicate that DMBT1 is more likely to exert anti-inflammatory effects in humans. The increase in DMBT1 expression in the murine colon compared to that in the jejunum could indicate that DMBT1 mainly exerts its functions in the murine colon, whereas doxorubicin mainly induces inflammatory changes in the small intestines. A previous study found that DMBT1 WT mice were partially protected against DSS-induced colitis^14^, which also suggests that DMBT1 is more likely to exert its functions in the murine colon.

Previous studies examining the role of DMBT1 in mice found that deletion of hensin/DMBT1 was lethal on embryonic day 4.5^37^ and that the conditional deletion of hensin/DMBT1 in intercalated cells resulted in distal renal tubular acidosis^38^, whereas the *Dmbt1*^*tm1KUMC*^ knockout strain did not show any effect on DSS-induced colitis^39^. For this study, we used DMBT1 knockout mice generated by Renner et al.^16^ that did not have any spontaneous phenotype and that previously had been shown to exhibit increased susceptibility to DSS-induced colitis. It is a limitation of this study that we only examined one mouse strain. However, since hensin/DMBT1 deletion is lethal in the embryonic stage and the *Dmbt1*^*tm1KUMC*^ strain showed no increased susceptibility to DSS-induced colitis, we believe that the strain produced by Renner et al. was the most appropriate for this study.

## Conclusion

We detected no significant effects of DMBT1 on chemotherapy-induced weight loss, inflammatory markers, functional enterocyte mass, or histologic inflammation three and seven days after doxorubicin treatment in mice. WT mice had significantly deeper crypts on D3 than DMBT1 KO mice. Doxorubicin induced a nonsignificant increase in jejunal and colonic DMBT1 expression.

## Methods

The mice were terminated prematurely according to humane endpoints if the weight loss exceeded 20% or if they showed a significant degree of pain. A total of five mice were prematurely terminated and excluded from the subsequent analysis due to reaching humane endpoints. These were three doxorubicin treated WT mice were terminated prior to endpoint due to weight loss > 20% (one at day 4 and two at day 5 and), one doxorubicin treated KO mouse was found dead at day 7, and one saline treated KO mouse was excluded due to injection error resulting in a necrotic kidney.

### Animals and genotyping procedure

DMBT1 C57BL/6 knockout (KO) mice generated by Renner et al.^16^ were kindly provided by Prof Jan Mollenhauer and bred in-house from heterozygous *Dmbt1*^+/−^ parental mice, and the offspring were cohoused in litters of mixed genotypes until one week before the start of the experiment.

Genotyping was performed on tail-tip biopsies both before and after the start of the experiment. DNA from tail biopsies was isolated using the DNeasy Blood & Tissue Kit (Qiagen, Hilden, Germany) according to the supplier’s instructions.

Seven days before the experiments, the mice were transferred from group housing into individual filter-top cages, allowing for acclimation to the new environment. Cages contained woodchip bedding, nesting material, tunnel housing, and a wooden chew. Food and water were supplied ad libitum. The mice were housed at 21–24 °C in 55% humidity and a 12-h light/dark cycle.

Ten- to 12-week-old female wild-type (WT) and KO littermates were included in the experiment.

### Mucositis induction and collection of samples

A total of 62 mice were randomized into eight treatment groups. Mucositis was induced with 20 mg/kg doxorubicin (Doxorubicin Accord, Accord Healthcare Limited, North Harrow, Middlesex, England) that was diluted 1:1 in isotonic saline and administered by two bilateral intraperitoneal injections. The control mice were injected with an equivalent volume of isotonic saline.

The following mice were included in the subsequent analysis: 20 mice (10 WT and 10 KO) received doxorubicin and were terminated three days after the injection (D3); 10 mice (5 WT and 5 KO) received saline and were terminated on D3; 22 mice (12 WT and 10 KO) received doxorubicin and were terminated seven days after the injection (D7); and 10 mice (5 WT and 5 KO) received saline and were terminated on D7. In all subsequent figures, the results for the saline groups from D3 and D7 were pooled into WT and KO groups.

Terminations were carried out by cardiac puncture under ketamine/xylazine anesthesia (100 mg/kg/10 mg/kg (MSD Animal Health, Copenhagen, Denmark/Recipharm Monts, France)) followed by cervical dislocation. Blood was stored at room temperature for at least two hours and centrifuged (2000 × *g*, 10 min, 4 °C), and the serum was separated and frozen at − 80 °C. After blood sampling, the heart was perfused with 10 mL of cold, sterile phosphate-buffered saline (PBS) (Sigma-Aldrich Chemie, Steinheim, Germany). The small intestine from the pyloric sphincter to the ileocecal transition was removed, measured, and cut into three equal-sized pieces representing the duodenal, jejunal, and ileal parts of the small intestine. The colon was also removed and measured. The distal ends of all segments were used for histological examination. The remaining segments were cut longitudinally, washed three times in 45 mL sterile PBS, snap-frozen in liquid nitrogen, and stored at -80 °C prior to further processing.

### Citrulline measurement

Serum levels of citrulline were measured and quantified with a UPLC-TQD MS (Waters, Milford, MA, USA) as described previously^41^ by a blinded researcher.

### Quantitative real-time PCR (qRT-PCR) of DMBT1, IL-1β, IL-6, and TNF

RNA was isolated from small intestinal and colonic tissue using Isol-RNA Lysis Reagent (5 Prime GmbH, Hamburg, Germany) according to the manufacturer’s instructions. RNA was analyzed using a NanoDrop (Thermo Scientific, Wilmington, DE, USA) to determine the concentration and purity. All RNA samples had an A260/A280 ratio > 1.8, suggesting adequate purity.

Complementary DNA (cDNA) was synthesized by an M-MLV Reverse Transcriptase kit (Sigma-Aldrich, Taufkirchen, Germany) according to the manufacturer's instructions. RT-PCR analysis was performed using TaqMan Universal Master mix II with no UNG (Applied Biosystems, Foster City, CA, USA) and TaqMan Gene Expression Assays for *DMBT1* (Mm00455996), *IL-1β (*Mm00434228), *IL-6* (Mm00446190), and *TNF* (Mm00443258) (Applied Biosystems, Foster City, CA, USA).

A pilot study was conducted to establish the appropriate endogenous control, and glyceraldehyde 3-phosphate dehydrogenase (*Gapdh,* Mm99999915) was selected due to its high and consistent expression in different groups.

Reactions were performed in duplicate by a blinded researcher using a StepOnePlus real-time PCR system (Applied Biosystems, Foster City, CA, USA). mRNA expression was determined relative to that of *Gapdh* using the 2^−ΔΔCt^ method and expressed as the fold change. qRT-PCR data were normalized to the data obtained from the WT Saline D3 or D7 group using the GeNorm method^42^.

### Histological preparation and evaluation

The samples for histological analysis were fixed for 24 h in 4% formaldehyde in aqueous solution (VWR chemicals, Leuven, Belgium), transferred to PBS with 0.05% NaN_3_ and stored at 4 °C until paraffin embedding and sectioning. The evaluation of tissue architecture and inflammatory activity was performed on H&E-stained tissue. The tissue was also stained for CD45 (Rat Anti-Mouse 30F11 antibody, BD Pharmingen, Albertslund, Denmark) and Ki67 (SP6, Rabbit Monoclonal Antibody, Cell Marque, Rocklin, California, USA), which aided in the evaluation of histologic damage, and for DMBT1 (GP340, rabbit polyclonal antibody, created in-house^17^. The histological evaluation was performed using three cross-sections obtained at 3 mm intervals from each intestinal segment. The H&E-stained slides were scanned and analyzed using NDP.view2 (NanoZoomer Digital Pathology, Hamamatsu Photonics, Japan).

The degree of intestinal inflammation, tissue damage, villous atrophy, and crypt hyperplasia were assessed using scoring methods for the small intestine modified from Kaczmarek et al.^43^ and de Koning et al.^44^. Small intestinal tissue was scored from 0 to 4 based on inflammatory cell infiltration and intestinal architecture, with a higher score representing increased severity. Villus height was measured from the villus-crypt junction to the villus tip in villi from a single layer of epithelial cells cut through the nuclei around the villus. Crypt depth was measured from the base of the crypt to the villus-crypt junction in crypts with open lumens and a continuous cell column on each side. Villus height and crypt depth were measured by a blinded researcher.

Colonic tissue was evaluated according to the method described in Kim et al.^45^. Scores ranged from 0 to 5 based on inflammatory cell infiltration, epithelial changes, and mucosal architecture. A higher score represented more severe damage. Scoring was based on H&E-stained tissue and either CD45-stained slides (which aided in the evaluation of inflammation) or Ki67-stained slides (which aided in the evaluation of epithelial hyperplasia). All histological evaluations were performed under supervision of by a blinded gastrointestinal pathologist.

### Statistical analysis

The Kruskal–Wallis test with Dunn’s correction was used to analyze weight loss, the levels of citrulline, expression of DMBT1, expression of proinflammatory cytokines, and histologic damage score. One-way ANOVA with Sidak’s test was used to analyze the intestinal length, villus height and crypt depth. Normality was determined using the Shapiro Wilks-W test and visually inspected using Q–Q-plots. The alpha level for all tests was 0.05. A *p*-value < 0.05 was considered significant. All data are presented as the mean ± standard error of the mean (SEM). All tests were performed with GraphPad Prism (version 8.3.1; GraphPad Software, La Jolla, CA).

### Ethical statement

All procedures were approved by the Danish National Committee on Animal Experimentation (J.no. 2014-15-0201-00417). All methods and procedures were performed in accordance with guidelines and regulations approved by the Danish National Committee on Animal Experimentation. The manuscript was prepared per the ARRIVE guidelines^40^.
